# Vitamin D protects against immobilization-induced muscle atrophy via neural crest-derived cells in mice

**DOI:** 10.1038/s41598-020-69021-y

**Published:** 2020-07-22

**Authors:** Satoshi Nakamura, Yuiko Sato, Tami Kobayashi, Yosuke Kaneko, Eri Ito, Tomoya Soma, Hiroyuki Okada, Kana Miyamoto, Akihito Oya, Morio Matsumoto, Masaya Nakamura, Arihiko Kanaji, Takeshi Miyamoto

**Affiliations:** 1 Department of Orthopedic Surgery, Keio University School of Medicine, 35 Shinano-machi, Shinjuku-ku, Tokyo, 160-8582 Japan; 2 Department of Advanced Therapy for Musculoskeletal Disorders II, Keio University School of Medicine, 35 Shinano-machi, Shinjuku-ku, Tokyo, 160-8582 Japan; 3 Department of Musculoskeletal Reconstruction and Regeneration Surgery, Keio University School of Medicine, 35 Shinano-machi, Shinjuku-ku, Tokyo, 160-8582 Japan; 4 Institute for Integrated Sports Medicine, Keio University School of Medicine, 35 Shinano-machi, Shinjuku-ku, Tokyo, 160-8582 Japan; 5 Division of Oral and Maxillofacial Surgery, Department of Dentistry and Oral Surgery, Keio University School of Medicine, 35 Shinano-machi, Shinjuku-ku, Tokyo, 160-8582 Japan; 6 Department of Orthopedic Surgery, The University of Tokyo, 7-3-1 Hongo, Bunkyo-ku, Tokyo, 113-0033 Japan; 7 Department of Orthopedic Surgery, Kumamoto University, 1-1-1 Honjo, Chuo-ku, Kumamoto, 860-8556 Japan

**Keywords:** Experimental models of disease, Endocrine system and metabolic diseases

## Abstract

Vitamin D deficiency is a recognized risk factor for sarcopenia development, but mechanisms underlying this outcome are unclear. Here, we show that low vitamin D status worsens immobilization-induced muscle atrophy in mice. Mice globally lacking vitamin D receptor (VDR) exhibited more severe muscle atrophy following limb immobilization than controls. Moreover, immobilization-induced muscle atrophy was worse in neural crest-specific than in skeletal muscle-specific VDR-deficient mice. *Tnfα* expression was significantly higher in immobilized muscle of VDR-deficient relative to control mice, and was significantly elevated in neural crest-specific but not muscle-specific VDR-deficient mice. Furthermore, muscle atrophy induced by limb immobilization in low vitamin D mice was significantly inhibited in Tnfα-deficient mice. We conclude that vitamin D antagonizes immobilization-induced muscle atrophy via VDR expressed in neural crest-derived cells.

## Introduction

An increase in the size of the elderly population in developed countries has provided a continuous and sharp increase in the number of patients with sarcopenia, frailty, osteoporosis and bone fragility fractures. Sarcopenia, which is marked by progressive reduction in muscle volume and power and/or decreased physical performance with age, causes reduced activities of daily living (ADL) and falls, leading to bone fragility fractures^1^. Low vitamin D status is reportedly common in sarcopenia patients^2,3^, and is often seen in osteoporosis patients with fragility fractures such as hip and vertebral fractures^4,5^. Several meta-analyses report that vitamin D activity can prevent falls^6–9^, and most hip fractures reportedly occur due to falls^10^. Taken together, these results suggest vitamin D deficiency is a risk for sarcopenia, falls and fragility fractures, although it remains unclear how vitamin D prevents these risks.

Vitamin D plays a pivotal role in calcium homeostasis, and either vitamin D deficiency or vitamin D receptor (VDR) mutation/deficiency promotes development of rickets in humans and mice^11–13^. The ubiquitously expressed transcription factor VDR stimulates expression of various genes in the presence of active vitamin D analogues such as 1,25(OH)_2_D_3_^14^. VDR activated by vitamin D stimulates expression of receptor activator of nuclear factor kappa b ligand (RANKL), a cytokine essential for osteoclast differentiation, and inhibits expression of osteoprotegerin (OPG) in osteoblastic cells, thereby promoting osteoclastogenesis indirectly via osteoblastic cells^15–17^. Moreover, vitamin D reportedly inhibits osteoclast differentiation induced by macrophage colony stimulating factor (M-CSF) and RANKL directly in osteoclast precursor cells via VDR^18,19^. VDR is required for intestinal calcium absorption, and intestine-specific VDR conditional knockout mice exhibit severe osteoporosis due to excessive calcium release from bones to augment circulating calcium^20^. Peripheral nerves and Schwann cells are also vitamin D targets, and insulin like growth factor 1 (IGF-1) expression is stimulated in sciatic nerve or cultured Schwann cells via VDR in the presence of vitamin D agonists^21^. The myelin protein zero (P0) is expressed in neural crest cells, which during development give rise to various cell types such as dorsal root ganglia and Schwann cells^22^, and P0 is also expressed in adult Schwann cells^23^. Thus, VDR deletion in P0-expressing cells promotes VDR deficiency in neural crest-derived cell types, including Schwann cells. VDR is also reportedly expressed in muscle and at the neuro-muscular junction^24^. Nonetheless, it remains unclear which cells are critical for regulation of muscle volume and power via VDR.

Muscle atrophy is promoted by many factors, including immobility and unloading^25^. Immobilization-induced muscle atrophy is promoted by denervation, fixation of a limb, or immobility and causes decreased muscle mass and power in both the elderly and young^26,27^. Immobilization reportedly reduces IGF-1 signaling in muscle, leading to accumulation of Smad2/3 protein levels^28^. Smad2/3 activities promote expression of the ubiquitin ligases *Atrogin-1* and *MuRF1*, leading to muscle protein degradation and muscle atrophy^28^.

Here, we show that vitamin D deficiency worsens immobilization-induced muscle atrophy in mice. Immobilization-induced muscle atrophy phenotypes were enhanced in neural crest-specific compared to skeletal muscle-specific VDR-deficient mice under vitamin D-sufficient conditions. Our study provides novel understandings on vitamin D functions to prevent bone fragility fractures or sarcopenia.

## Results

### Vitamin D deficiency worsens immobilization-induced muscle atrophy

**Figure 1:**
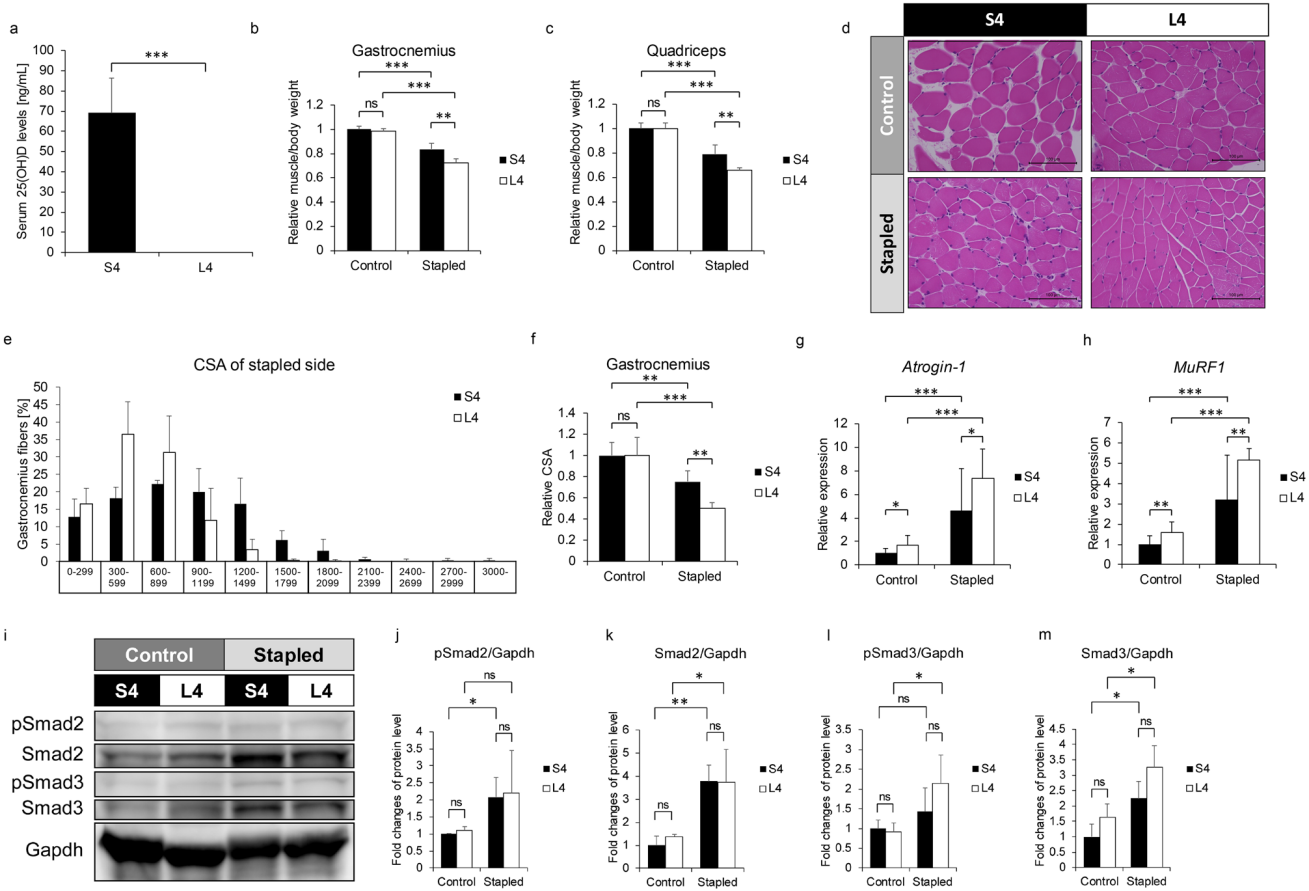
Vitamin D deficiency worsens immobilization-induced skeletal muscle atrophy. 6-week-old C57BK/6 female mice were fed a low (L) or standard (S) vitamin D diet for 4 weeks (L4 or S4 groups). Their left hind limbs were stapled at 9-weeks of age and mice were sacrificed 1 week after stapling. (**a**) Sera were collected at sacrifice (each group n = 5). Serum 25(OH)D levels were analyzed by radio immunoassay. (**b**, **c**) Wet weights of gastrocnemius (**b**) and quadriceps (**c**) muscles adjusted to body weight in the stapled versus the control side in S4 and L4 groups. Representative data of 2 independent experiments are shown. (**d**) Hematoxylin and eosin staining of gastrocnemius muscles in control and stapled sides of S4 and L4 groups. Scale bar, 100 µm. (**e**) Frequency distribution of fiber area of gastrocnemius muscles in the stapled side of S4 and L4 groups (x axis, fiber area; y axis, % of cross-sectional area (CSA) of muscle fiber; data represent mean % CSA ± SD). (**f**) Relative mean cross-sectional areas (CSA) of gastrocnemius muscles on control and stapled sides of S4 and L4 groups. (**g**–**h**) Relative *Atrogin-1* (**g**) and *MuRF1* (**h**) expression in control and stapled gastrocnemius muscles of S4 and L4 groups based on quantitative realtime PCR. (**i**) Western blot of Smad2 and 3 protein in control and stapled gastrocnemius muscles of S4 and L4 groups. Representative images are shown. (**j**–**m**) Quantitation of levels of phosphorylated Smad2 (**j**), total Smad2 (**k**), phosphorylated Smad3 (**l**) and total Smad3 (**m**) per Gapdh shown as means ± SD relative to control side of S4 and L4 groups (S4, n = 3; L4, n = 3). (**b**, **c** and **f**) Means ± SD relative to control side of S4 group are shown. (**g** and **h**) Shown is mean indicated expression relative to *Gapdh* ± SD relative to control side of S4 group. Statistical analysis was done by Student’s t-test (*P < 0.05; **P < 0.01; ***P < 0.001; ns, not significant).

To establish vitamin D-deficient conditions, we fed 6-week-old wild-type (WT) mice a low (L) or standard (S) vitamin D diet for 4 weeks (Fig. 1) and then monitored serum 25(OH)D levels (Fig. 1a). Mice fed the L diet showed significantly reduced serum 25(OH)D levels compared with mice fed the standard vitamin D diet (Fig. 1a). We then created an immobilization-induced muscle atrophy model in lower extremities by stapling of hind limbs, as previously described^28^. Specifically, at 6-weeks of age, we began feeding WT mice the L or S diet, three weeks later immobilized the hind limb, and a week after that sacrificed animals and analyzed muscle volume in gastrocnemius and quadriceps. Volume of both muscles decreased following immobilization, and that effect was significantly more severe in mice fed a low (L4) rather than standard (S4) diet (Fig. 1b, c). Histological analysis showed that cross sectional area (CSA) of gastrocnemius muscle in mice fed the L diet was significantly smaller than in mice fed the S diet (Fig. 1d–f). Moreover, expression of the atrogenes *Atrogin-1* and *MuRF1*, both E3 ubiquitin ligases, was significantly higher in gastrocnemius muscle of L versus S mice (Fig. 1g, h). Increased levels of Smad2 and Smad3 proteins, upstreams of the atrogenes, are reportedly required for immobilization-induced muscle atrophy^28^. Indeed, Smad2/3 protein expression increased following immobilization in gastrocnemius muscle compared with non-stapled side (Fig. 1i–m).

### Refeeding a standard diet or administration of a vitamin D agonist rescues muscle atrophy phenotypes in limb-immobilized vitamin D-deficient mice

**Figure 2:**
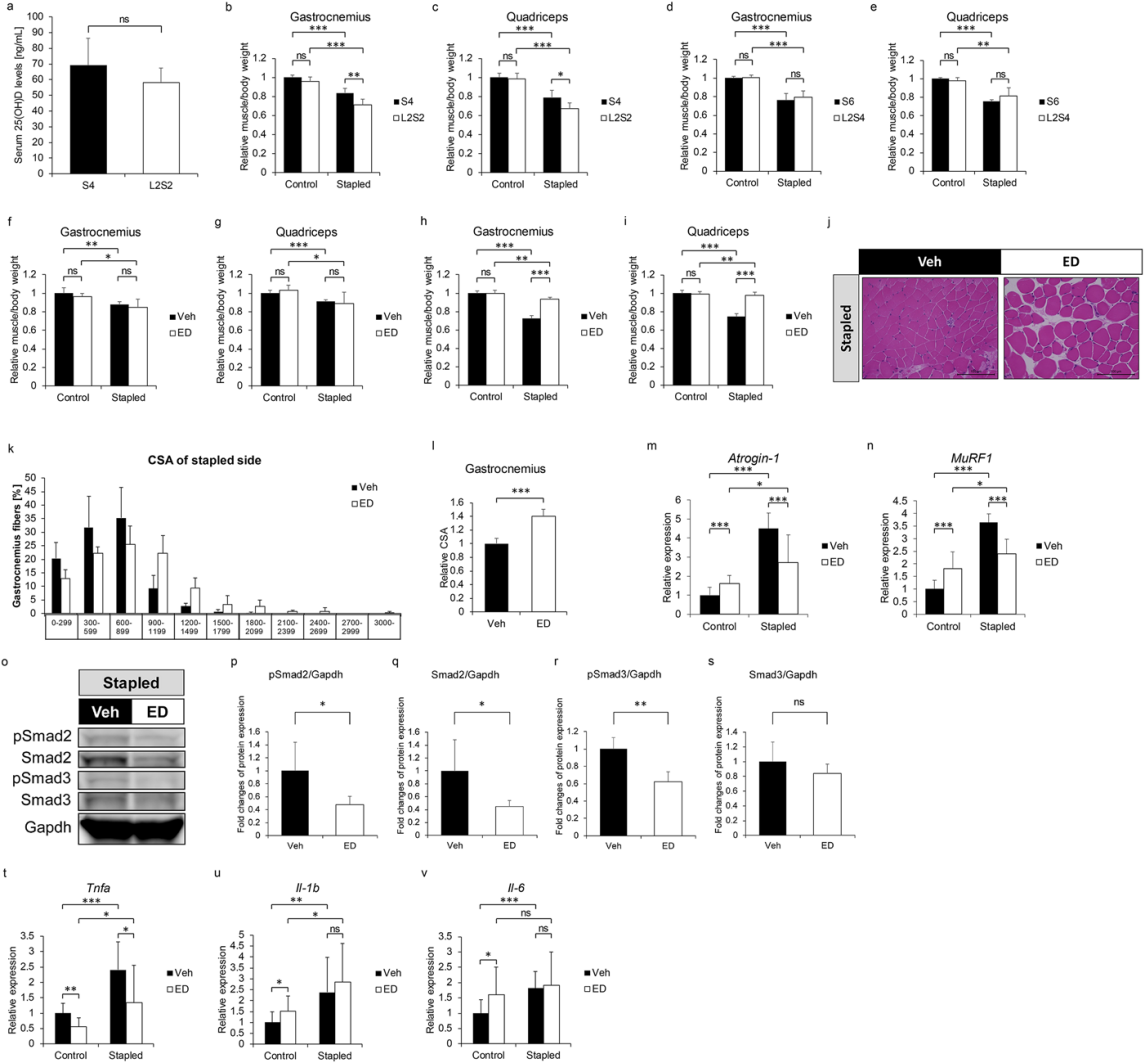
Refeeding a normal vitamin D diet or treatment with a vitamin D analogue rescues atrophy phenotypes following muscle immobilization. (**a**–**c**) 6-week-old female WT mice were fed the L diet for 2 weeks and then the S diet for 2 weeks (L2S2 group), or fed the S diet for 4 weeks (S4 group). Hind limbs were stapled at 9-weeks of age during the diet regime. Mice were sacrificed 1 week after stapling, and Sera were collected (Each group n = 5). (**a**) Serum 25(OH)D levels in indicated groups were analyzed by radioimmunoassay. (**b**, **c**) Weights of gastrocnemius (**b**) and quadriceps (**c**) muscles adjusted to body weight relative to those of control sides of the S4 and L2S2 groups. (**d**, **e**) 6-week-old female WT mice were fed the L diet for 2 weeks and then the S diet for 4 weeks (L2S4 group), or fed the S diet for 6 weeks (S6 group). Left hind limbs were stapled at 11-weeks of age and mice were sacrificed 1 week after stapling (each group n = 5). Wet weights of gastrocnemius (**d**) and quadriceps (**e**) muscles adjusted to body weight of S6 and L2S4 groups relative to control sides of the S6 group. (**f**–**v**) 6-week-old female WT mice were fed the S (**f** and **g**) or L (**h**–**v**) diet, and treated with 3.5 ng ED71 (ED group) or vehicle (Veh group) twice per week starting at 8 weeks of age. Left hind limbs stapled at 9 weeks of age and mice were sacrificed a week later (each group n = 5). (**f**–**i**) Wet weights of gastrocnemius (**f** and **h**) and quadriceps (**g** and **i**) adjust to body weight in Veh and ED groups fed the S (**f** and **g**) or L (**h** and **i**) diet. (**j**) Hematoxylin and eosin staining of gastrocnemius muscles on stapled side of indicated groups fed an L diet. Scale bar, 100 µm. (**k**) Frequency distribution of fiber area of gastrocnemius muscles on stapled side of indicated groups fed the L diet (**h**; x axis, fiber area; y axis, % of cross-sectional area (CSA) of muscle fiber; data are mean % of CSA ± SD). (**l**) Relative mean CSA. (**m**, **n**) Relative *Atrogin-1* (**m**) and *MuRF1* (**n**) expression in control and stapled gastrocnemius muscles of indicated group fed the L diet as analyzed by quantitative realtime PCR. (**o**) Smad2 and 3 total and phosphorylated protein levels in stapled gastrocnemius muscles of indicated groups fed the L diet, based on western blotting. Representative images are shown. (**p**–**s**) Quantitation of data shown in (**o**). (**t**–**v**) Relative expression of indicated cytokines in control and stapled gastrocnemius muscles of Veh and ED group fed the L diet, based on quantitative realtime PCR. (**b**–**e** and **f–i**) Means ± SD were shown relative to control side of S4 (**b**–**e**) or Veh (**f**–**i**) group. (**m**, **n** and **t**–**v**) Shown is mean indicated expression relative to *Gapdh* ± SD relative to control side of Veh group. (**p**–**s**) Shown is mean indicated protein levels relative to Gapdh ± SD relative to the Veh group. Statistical analysis was done by Student’s t-test (*P < 0.05; **P < 0.01; ***P < 0.001; ns, not significant). (**b**–**i**, **m**, **n** and **t**–**v**) Shown are representative data of 2 independent experiments.

We next varied the protocol used to establish vitamin D-deficient mice by feeding 6-week-old WT mice either the S diet for 4 or 6 weeks (S4 or S6) or the L diet for 2 weeks followed by the S diet for either 2 or 4 more weeks (L2S2 or L2S4) (Figs. S1a, b and 2a–e) and then monitored serum 25(OH)D levels. Serum 25(OH)D levels of L2S2 group were equivalent to S4 group (Fig. 2a). On the other hand, when we applied this protocol and immobilized the hind limb at week 9 (S4 or L2S2) (Fig. S1a) or 11 (S6 or L2S4) (Fig. S1b), we observed rescue of immobilization-induced phenotypes in both muscles based on muscle weight in the L2S4 but not the L2S2 group (Fig. 2b–e). Moreover, administration of the vitamin D analogue, eldecalcitol, at 8-weeks of age did not alter volume of either muscle in an immobilization model in mice fed the S diet only (Figs. S1c and 2f and g). However, we observed significant rescue of immobilization-induced muscle atrophy in gastrocnemius and quadriceps of 10-week old mice that had been administered eldecalcitol for two weeks and fed the L diet starting at 6 weeks of age (Fig. 2h and i). CSA in gastrocnemius muscle of eldecalcitol-treated mice was also significantly larger than that in vehicle-treated mice (Fig. 2j–l). Moreover, higher *Atrogin-1* and *MuRF1* expression in immobilized gastrocnemius was significantly inhibited by eldecalcitol treatment for 2 weeks (Fig. 2m and n). Similarly, elevated pSmad2/3 levels seen in immobilized gastrocnemius were attenuated by eldecalcitol treatment (Fig. 2o–s). Finally, eldecalcitol treatment for 2 weeks significantly blocked upregulation of *tumor necrosis factor alpha* (*Tnfα*) promoted by immobilization (Fig. 2t–v).

### VDR-deficient mice exhibit muscle atrophy without immobilization

**Figure 3:**
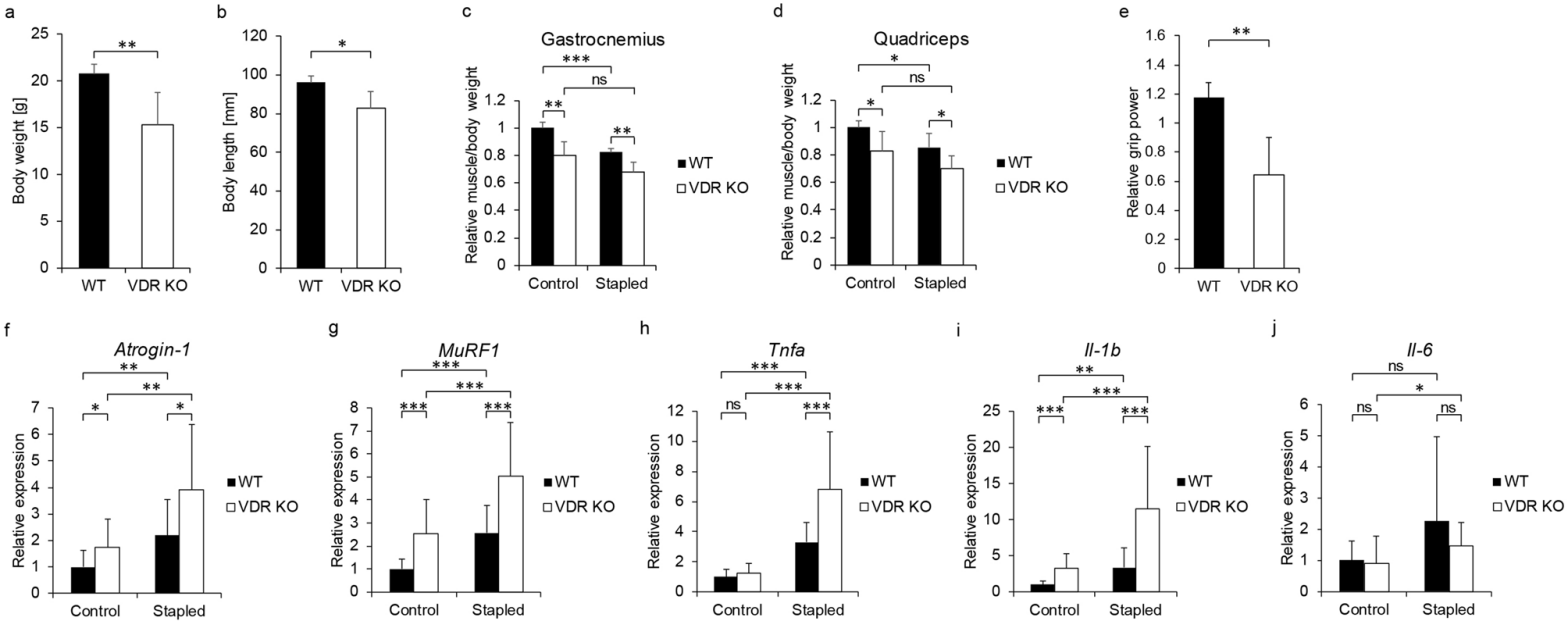
VDR KO mice exhibit worsened atrophy after immobilization and elevated expression of *Atrogin-1*, *MuRF1*, *Tnfa* and *Il-1b*. VDR KO or WT mice were fed a high calcium diet after weaning, their left hind limbs were stapled at 9 weeks of age, and animals were sacrificed 1 week later (each mouse n = 5). (**a**, **b**) Body weight (**a**) and length (**b**) of WT and VDR KO mice at sacrifice. (**c**, **d**) Wet weights of gastrocnemius (**c**) and quadriceps (**d**) muscles adjusted to body weight on control and stapled sides of WT and VDR KO mice relative to values on WT control side. (**e**) Forelimb grip power of VDR KO mice relative to WT mice. (**g**–**i**) Relative *Atrogin-1* (**f**), *MuRF1* (**g**), *Tnfa* (**h**), *Il-1b* (**i**) and *Il-6* (**j**) expression in control and stapled gastrocnemius muscles of indicated mice relative to WT control side, as determined by quantitative realtime PCR (each mouse n = 6). (**c**, **d**) means ± SD relative to control side of WT mice are shown. (**e**) mean ± SD relative to WT mice is shown. (**f–j**) Mean expression relative to *Gapdh* ± SD relative to WT mice is shown. Statistical analysis was done by Student’s t-test (*P < 0.05; **P < 0.01; ***P < 0.001; ns, not significant). (**a**–**e**) Representative data of 2 independent experiments.

We next analyzed muscle atrophy in global Vdr knockout (Vdr KO) and WT mice, and immobilized the hind limb at week 9 (Fig. 3). VDR deficient mice were smaller than WT mice at 10 weeks of age when the mice were sacrificed (Fig. 3a, b), and muscle weight per body weight of both gastrocnemius and quadriceps in the non-stapled side was significantly lower in VDR KO relative to WT mice (Fig. 3c, d). Grip power was also significantly lower in VDR KO relative to WT mice (Fig. 3e).

Immobilization-induced atrophy of both gastrocnemius and quadriceps muscle was comparable in VDR KO and WT mice (Fig. 3c, d). However, *Atrogin-1* and *MuRF1* expression following immobilization was significantly higher in gastrocnemius of VDR KO compared to WT mice (Fig. 3f, g). Furthermore, induction of *Tnfα* or *interleukin-1 beta (IL-1β)* in gastrocnemius muscle following immobilization was significantly higher in VDR KO compared to WT mice (Fig. 3h–j).

### VDR expressed in neural crest-derived rather than skeletal muscle cells antagonizes immobilization-induced muscle atrophy

**Figure 4:**
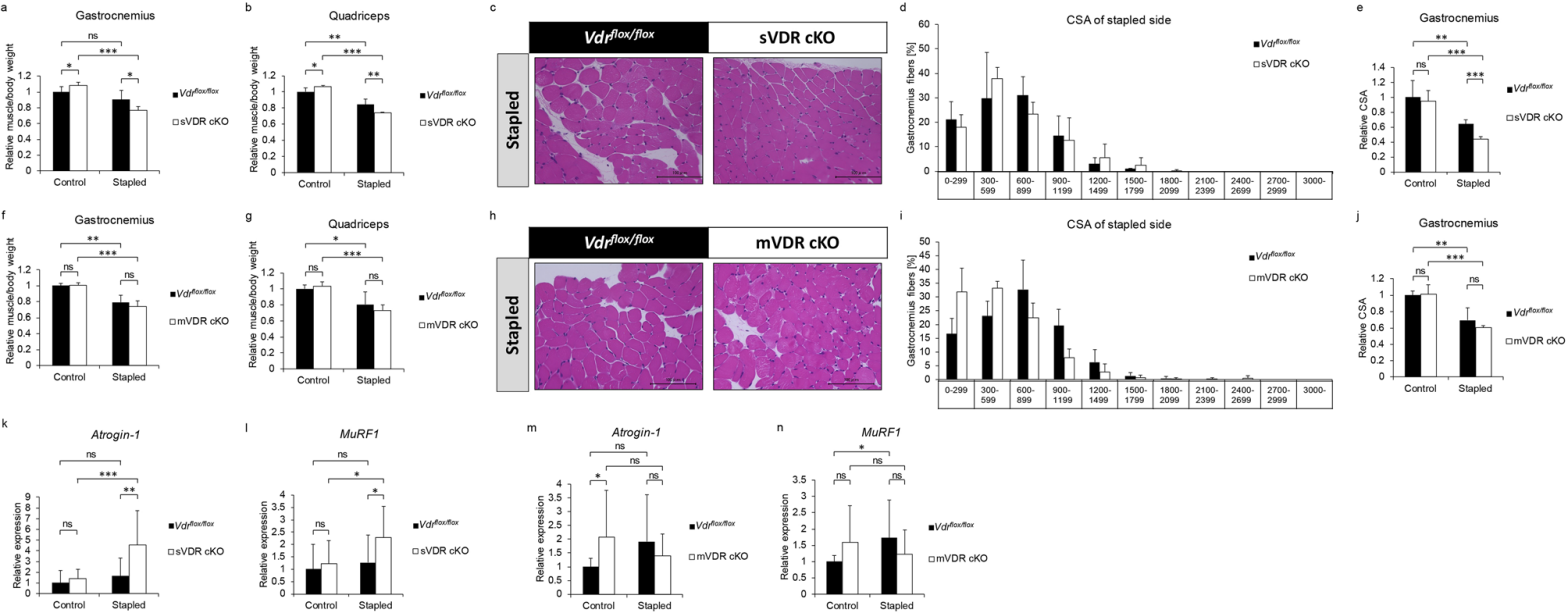
Vitamin D attenuates immobilization-induced muscle atrophy through VDR expressed in neural crest-derived cells. Neural-crest-specific sVDR cKO (P0 Cre;*VDR*^*flox/flox*^), skeletal-muscle-specific mVDR KO (CkmmCre;*Vdr*^*flox/flox*^) and control (*Vdr*^*flox/flox*^) female mice were fed the S diet, their hind limbs were stapled at 9 weeks of age, and they were sacrificed 1 week later. (**a**–**e**, **f**–**j**) Wet weights of gastrocnemius (**a**, **f**) and quadriceps (**b**, **g**) muscles adjusted to body weight in control and stapled sides of sVDR cKO (**a**, **b**), mVDR cKO (**f**, **g**) or *Vdr*^*flox/flox*^ mice (**a**, **b**, **f** and **g**) relative to the control side of *Vdr*^*flox/flox*^ mice. Hematoxylin and eosin staining (**c**, **h**), frequency distribution of fiber area (**d**, **i**) and relative mean CSA (**e**, **j**) of gastrocnemius muscles in stapled side of sVDR cKO (**c**–**e**), mVDR cKO (**h**–**j**) and *Vdr*^*flox/flox*^ mice (**c**, **h**; Scale bar, 100 µm. **d**, **i**; x axis, fiber area; y axis, % of cross-sectional area (CSA) of muscle fiber; data are mean % of CSA ± SD). (**k**-**n**) Relative *Atrogin-1* (**k**, **m**) or *MuRF1* (**l**, **n**) expression in control or stapled gastrocnemius muscles of indicated genotypes. (**a**, **b**, **e**–**g** and **j**) Means ± SD of sVDR cKO (**a**, **b** and **e**), mVDR cKO (**f**, **g** and **j**) or *Vdr*^*flox/flox*^ mice relative to those in control side of *Vdr*^*flox/flox*^ mice. (**k**–**n**) Mean expression relative to *Gapdh* ± SD of sVDR cKO (**k** and **l**), mVDR cKO (**m**, **n**) or *Vdr*^*flox/flox*^ mice relative to *Vdr*^*flox/flox*^ mice is shown. Statistical analysis was done by Student’s t-test (*P < 0.05; **P < 0.01; ***P < 0.001; ns, not significant). (**a**, **b**, **f**, **g**, **m** and **n**) Representative data of 2 independent experiments are shown.

**Figure 5:**
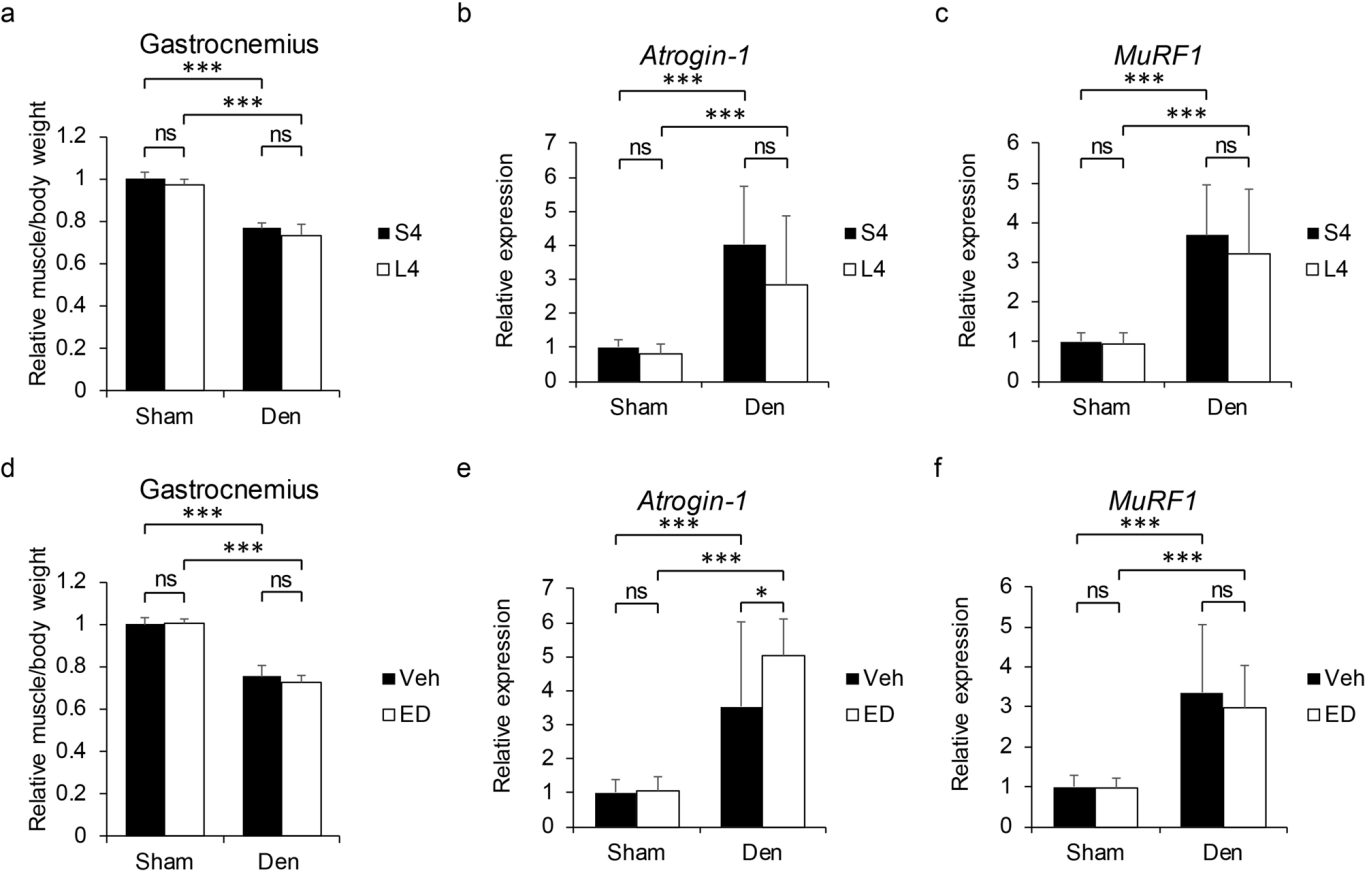
Denervation-induced skeletal muscle atrophy is not affected by vitamin D deficiency and vitamin D analogue treatment. 6-week-old C57BK/6 female mice were fed a standard (S) or low (L) vitamin D diet for 4 weeks (S4 or L4 group), and treated with 3.5 ng ED71 (ED group) or vehicle (Veh group) by intraperitoneal injection 2 times per week from 8-week old. Their left sciatic nerve was cut, and 1-mm portion was removed to denervate gastrocnemius muscle at 9-week of age. Their right hind limb was receipted only skin incision as sham surgery. They were sacrificed 1 week after surgery. (each group n = 5). (**a**, **d**) Wet weights of gastrocnemius muscles adjusted with body weight of S4 or L4 group with vehicle injection (**a**), and L4 group with vehicle or ED71 injection (**d**). (**b**, **c**, **e**, **f**) Relative expressions of *Atrogin-1* (**b**, **e**) and *MuRF1* (**c** and **f**) in control (sham) and denervated gastrocnemius muscles of S4 or L4 group with vehicle injection (**b**, **c**), and L4 with vehicle or ED71 injection (**e**, **f**). Mean ± SD relative to sham side of S4 (**a**–**c**) or Veh (**d**–**f**) group are shown. Statistical analysis was done by Student’s t-test (*P < 0.05; **P < 0.01; ***P < 0.001; ns, not significant).

VDR is reportedly expressed in various cell types such as peripheral nerve, Schwann and muscle cells^24,29–31^; among these, Schwann cells are derived from neural crest cells^32^. To determine which cells are critical in regulating immobilization-induced muscle atrophy via VDR, we established 2 lines of conditional VDR knockout mice. In one, we crossed *VDR*^*flox/flox*^ mice with neural crest cell-specific P0 Cre mice to yield P0 Cre;*VDR*^*flox/flox*^ mice (or, sVDR cKO). In the other, we established skeletal muscle-specific conditional VDR KO mice using the muscle creatine kinase promoter to generate Ckmm Cre; *VDR*^*flox/flox*^ mice (or, mVDR cKO). Immunohistochemical analysis of both lines revealed loss of VDR protein expression in P0-positive Schwann cells (Fig. S2a) and laminin-positive muscle fibers (Fig. S2b) in respective sVDR cKO and mVDR cKO mice fed an S diet. Then, when mice reached 9 weeks of age, we applied the immobilization protocol to both sVDR and mVDR cKO animals and 1 week later evaluated muscle phenotypes in cKO and corresponding control mice. Immobilization-induced atrophy in both gastrocnemius and quadriceps was significantly more severe in sVDR cKO than in control mice (*VDR*^*flox/flox*^) fed the S diet (Fig. 4a, b). Histological and CSA analysis confirmed that immobilization-induced atrophy was significantly more severe in sVDR cKO than in control mice (Fig. 4c–e). By contrast, immobilization-induced gastrocnemius and quadriceps atrophy was comparable in mVDR cKO and corresponding control mice (Fig. 4f–j). *Atrogin-1* and *MuRF1* expression in immobilized gastrocnemius was also significantly higher in sVDR cKO than in control mice (Fig. 4k, l), but those levels were comparable in mVDR and control mice (Fig. 4m, n). Neural crest-derived cells give rise to various tissue types, among them adrenal glands, which regulate muscle homeostasis^33^. Adrenal grands produce the glucocorticoid cortisol, which promotes muscle atrophy^34^. However, serum cortisol levels were comparable in sVDR cKO and control mice (Fig. S3a). Neural crest cells also contribute to formation of the parathyroid gland^35^; however, serum levels of parathyroid hormone were equivalent in sVDR cKO and control mice (Fig. S3b). Moreover, ED71 treatment did not rescue immobilization-induced muscle atrophy in either gastrocnemius or quadriceps muscle of sVDR mice (Fig. S4). These results suggest that VDR expressed in neural crest-derived cells, antagonizes immobilization-induced muscle atrophy in the presence of active vitamin D analogues. Indeed, when we established an immobilization model in WT mice that had been subjected to denervation of gastrocnemius muscle by cutting the sciatic nerve at 9 weeks of age, we observed comparable levels of muscle atrophy and atrogene expression in model mice fed the L or S diets (Fig. 5a–c) or in vitamin D-deficient mice treated with ED71 or vehicle (Fig. 5d–f). These results suggest overall that sciatic nerve loss impairs the ability of vitamin D to antagonize immobilization-induced muscle atrophy, likely via effects on Schwann cells.

**Figure 6:**
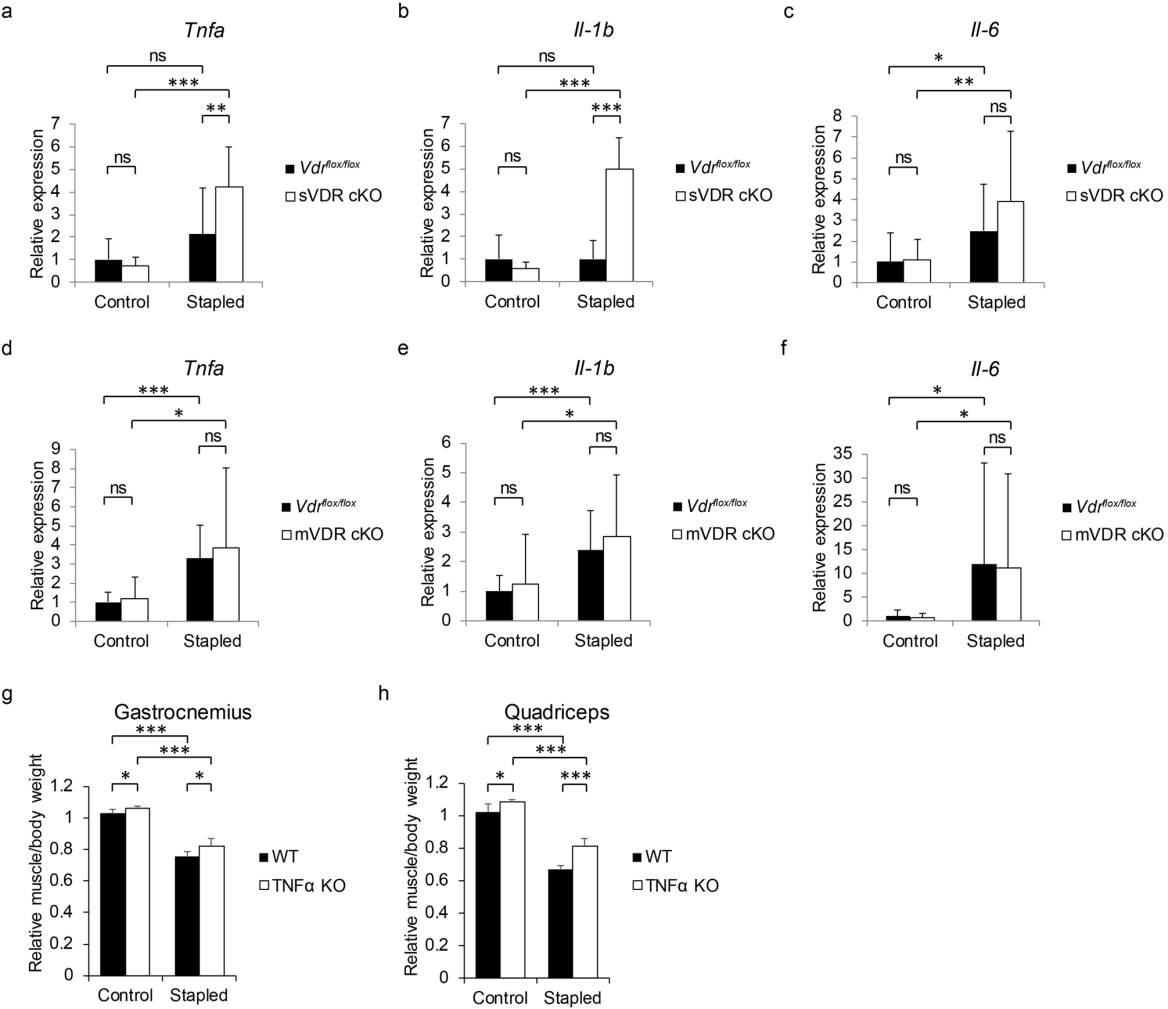
VDR in neural crest derived cells protects skeletal muscles from increased levels of *Tnfa* expression and immobilization-induced atrophy. (**a**-**f**) sVDR (neural-crest-specific) or mVDR (skeletal-muscle-specific) KO or control (*Vdr*^*flox/flox*^) female mice were fed the S diet, their hind limbs stapled at 9 weeks of age, and mice were sacrificed 1 week after stapling for analysis of expression of *Tnfa* (**a**, **d**), *Il-b* (**b**, **e**) and *Il-6* (**c**, **f**) relative to *Gapdh* in gastrocnemius muscles of indicated genotypes. (**g**, **h**) 6-week-old female TNFα KO (*Tnfα*^-/-^) or WT mice were fed the L diet and subjected to the same protocol as described above (each group n = 5). Wet weights of gastrocnemius (**g**) and quadriceps (**h**) muscles adjusted to body weight in control and stapled sides of TNFα KO and WT mice were determined relative to values on the WT control side. (**a**–**h**) Mean indicated parameters in sVDR cKO, mVDR cKO, *Vdr*^*flox/flox*^ (**a**–**f**), TNFα KO or WT (**g** and **h**) mice ± SD relative to those on the stapled side of *Vdr*^*flox/flox*^ (**a**–**f**) of WT (**g** and **h**) mice are shown. Statistical analysis was done by Student’s t-test (*P < 0.05; **P < 0.01; ***P < 0.001; ns, not significant).

mVDR or sVDR cKO mice fed the S diet were applied the immobilization protocol at nine weeks of age, and one week later, expression of the inflammatory cytokines *Tnfα* and *IL-1β* in immobilized gastrocnemius muscle was analyzed (Fig. 6a–f). We found that that expression was significantly higher in sVDR cKO than control mice (Fig. 6a–c) but differences in these levels were not significant in mVDR cKO versus control muscle (Fig. 6d–f). Finally, *Tnfα*^*−/−*^ (TNFα KO) mice fed the L diet from 6 weeks of age were subjected to limb immobilization at 9 weeks old. One week later, Tnfα KO mice showed a partial but significant rescue of atrophy of gastrocnemius or quadriceps muscle (Fig. 6g, h).

## Discussion

Vitamin D plays diverse roles in calcium, bone and muscle homeostasis as well as in IGF-1 induction^36^. Most IGF-1 is secreted from the liver, and serum 25 (OH) D levels correlate positively with serum IGF-1 levels^37^. Vitamin D also stimulates local IGF-1 production^38^, and indeed, here we show induction of IGF-1 expression in sciatic nerve from control (*VDR*^*flox/flox*^) mice, an effect absent in sVDR cKO mice (Fig. S5). We previously showed that IGF-1 treatment suppressed Smad2/3 activation in muscle cells in vitro^28^. Others have reported that IGF-1 suppresses inflammatory cytokine expression in liver and in atherosclerotic plaque of aortic sinuses^39,40^. Thus decreased local IGF-1 levels due to VDR loss in neural crest-derived cells likely promote local inflammation, worsening immobilization-induced muscle atrophy in sVDR cKO mice. In mammals, synthesis of most circulating vitamin D is stimulated in skin by sun exposure, but some is also consumed in the diet^41,42^. Elderly people tend to spend longer periods indoors, limiting sun exposure and potentially leading to vitamin D deficiency. Accordingly, vitamin D deficiency is frequently seen in the elderly^43,44^. However, young people are equally vulnerable to low vitamin D status^45–47^, making vitamin D deficiency a general concern.

**Figure 7:**
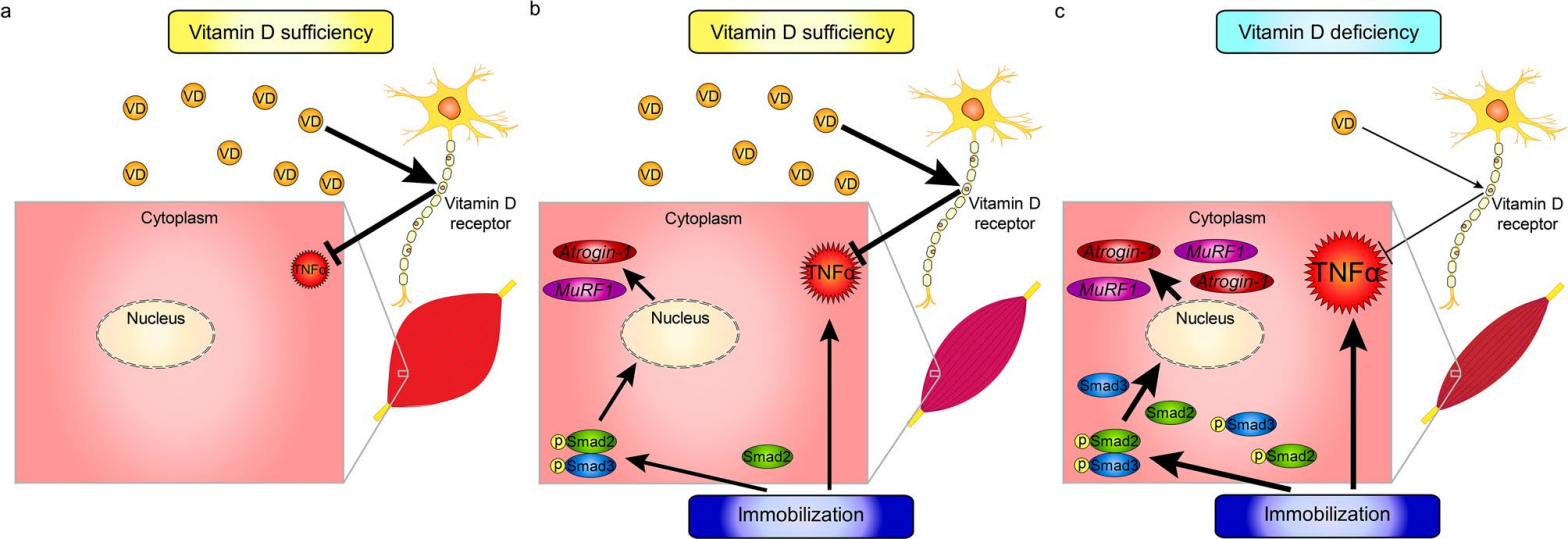
Model showing relationship of vitamin D activity to immobilization-induced skeletal muscle atrophy. Depicted is (**a**) the normal state in conditions of vitamin D sufficiency, (**b**) that same state subjected to immobilization, and (**c**) immobilization in the presence of a vitamin D-deficient state.

Here, we use a mouse model to show that muscle atrophy seen following limb immobilization is more severe under vitamin D-deficient conditions (Fig. 7). Our findings overall suggest that immobilization promotes local inflammatory cytokine expression in skeletal muscles via neural crest-derived cells under vitamin D deficient conditions, leading to severe muscle atrophy (Fig. 7). Our findings suggest that maintaining appropriate vitamin D levels is crucial to protect muscle from significant atrophy.

Various biological function of vitamin D have been reported^36^, but overall, reports of manifestations of vitamin D or VDR deficiency in humans are limited development of rickets or alopecia. VDR-deficient mice die after weaning, a lethality reportedly rescued by feeding a high calcium diet^13,48^ suggesting that vitamin D-VDR signaling is required to maintain calcium homeostasis. However, the impact of vitamin D activity on preventing falls is not as striking as its impact on inhibiting rickets development or regulating calcium homeostasis. Thus, prevention of falls is likely provided by a combination of several minor biological functions of vitamin D.

Falls in the elderly frequently occur due to impaired balance, and administration of an active vitamin D analogue reportedly improves balance in the elderly^49–51^. Indeed, administration of an active vitamin D analogue to osteoporosis patients was shown to significantly reduce the frequency of forearm fractures^52,53^, most of which occur by falls. Myoblasts and myotubes are known to express VDR and CYP27B1 (also called 1α-hydroxylase), which converts 25(OH)D to the active vitamin D3, 1,25(OH)_2_D_3_^24,54,55^. These results suggest that active vitamin D analogues directly act on muscle cells via VDR, or that muscle cells can convert inactive 25(OH)D to active 1,25(OH)_2_D_3_ by CYP27B1 activity in an autocrine or paracrine manner. Indeed, an active vitamin D analogue reportedly promotes muscle cell differentiation^56^. VDR signaling also functions in regulating neuromuscular maintenance and enhances locomotor ability^38^. However, our current study strongly suggests that muscle atrophy due to vitamin D deficiency has its origins in neural crest-derived rather than skeletal muscle cells.

Vitamin D promotes expression of IGF-1^57^, which acts as an anabolic factor and an inhibitor of catabolic signals in muscles^28,58,59^. Thus, IGF-1 plays a pivotal role in maintaining muscle homeostasis. Serum IGF-1 levels reportedly decrease with age in humans^60^, and reducing IGF-1 levels in adult mice promotes muscle atrophy and reduces muscle power, phenotypes seen in sarcopenia patients^61^. Peripheral nerve dysfunction also causes falls^62^, and proper function of peripheral nerves is determined by neurons and Schwann cells, which are required for peripheral nerve myelination. Schwann cells are known targets of vitamin D, and IGF-1 expression is stimulated in Schwann cells by vitamin D^21^. Our data suggests that vitamin D inhibits immobilization-induced muscle atrophy in part by inhibiting local inflammation and that vitamin D may prevent falls by a combination of these functions.

Vitamin D levels are regulated by various pathways. Vitamin D whether synthesized in skin or taken through the diet is converted into 25(OH)D in liver and stored^63^. Vitamin D levels in individuals are evaluated by serum 25(OH)D concentration, and levels < 20 ng/ml are defined as vitamin D deficiency, while those > 30 ng/ml are desirable^43,64^. 25(OH)D is converted into 1,25(OH)_2_D_3_, which promotes calcium and phosphorus absorption from the intestine^65^ and inhibits PTH expression. Elevated serum phosphorus levels stimulate FGF23 expression in osteocytes, which then inhibits *Cyp27b1* expression via the FGF receptor/Klotho co-receptor in kidney, downregulating 1,25(OH)_2_D_3_ levels via a negative feedback loop. Ectonucleotide pyrophosphatase/phosphodiesterase (Enpp1) also reportedly regulates serum 1,25(OH)_2_D_3_ levels by regulating *Klotho* expression under phosphate overload conditions^66^. Overall, either loss of or excessive activation of these axes deregulates serum vitamin D levels.

In summary, we show that low vitamin D status worsens muscle atrophy. The increasing number of sarcopenia patients and individuals showing low vitamin D status is a global concern, and these conditions are frequently associated with each other. Our data suggests that maintaining vitamin D status at an appropriate level before injury or decline in physical activity is likely crucial to prevent deterioration and muscle atrophy.

## Materials and methods

### Mice

C57BL/6 (WT) mice were purchased from Sankyo laboratory. *Vdr*^*-/-*^ (VDR KO) mice were established previously^13^. *CkmmCre/Vdr*^*flox/flox*^ (mVDR cKO) mice and *P0Cre/Vdr*^*flox/flox*^ (sVDR cKO) mice were obtained by mating skeletal-muscle specific *CKmmCre* mice (The Jackson Laboratory) and neural-crest specific *P0Cre* mice (Kumamoto University) with *Vdr*^*flox/flox*^ mice^67^. *Vdr*^*flox/flox*^ mice served as controls. All mice were maintained under specific pathogen-free conditions and kept under a 12-h light/dark cycle and fed a standard (S) diet, a low Vitamin D (L) diet or *high calcium (HC) diet*. The L diet contains vitamin D3 and Ca: 0.47%, P: 0.3% (T-17368, Crea, Japan) and a HC diet contains Ca: 2%, P: 1.25% (T-15472, Crea, Japan). All animal experiments were carried out in accordance with the Institutional Guidelines on Animal Experimentation at Keio University, and animal experiment protocols were approved by the Keio University Institutional Animal Care and Use Committee.

### Hind limb stapling

Midfoot parts of left hind limbs were fixed with the maximum flexion position of hip, knee and ankle joints to back skins pulled down to the feet by AUTOCLIP 9 mm and CLIP Applier. Right hind limbs served as controls and were not stapled.

### Denervation

Nine-week old mice were anesthetized. Their left sciatic nerves were cut, and 1-mm portion of them was removed to denervate gastrocnemius muscle. Their right hind limbs were receipted a skin incision as sham surgery.

### ED71 treatment

Mice were injected with 3.5 ng ED71 or vehicle (ethanol) intraperitoneally 2 times per week from 8-weeks of age. The protocol was: days 1 and 4, injection; day 5, stapling; days 8 and 11, injection; and day 12, sacrifice.

### Grip power measurement

Grip power of fore limbs was measured immediately before sacrifice. Mice held a wire netting (MK380Si, Muromachi Kikai Co., Tokyo, Japan) with only their forearms, and their tails were pulled backwards horizontally. Maximal pulling power, or grip power, was judged when mice released the wire and was measured 10 times per mouse.

### Histology

Gastrocnemius muscles were embedded into paraffin, cut into 4 μm sections and stained with hematoxylin and eosin. Fiber cross-sectional area (CSA) was analyzed with BioRevo (Keyence, Osaka, Japan). The following number of myofibers per mouse per group: S4, 62–102; L4, 58–158; Veh, 122–148; ED, 86–109; *Vdr*^*flox/flox*^, 54–121; sVDR cKO, 55–179; and mVDR cKO. 55–138.

### Blood tests

Sera were collected immediately before sacrifice. Serum 25(OH)D levels were analyzed by radioimmunoassay.

### Quantitative realtime PCR

Total RNA was isolated from gastrocnemius muscle using TRI Reagent (TR118, Molecular Research Center, Inc., Cincinnati, OH) and an RNeasy Mini Kit (74106, QIAGEN, Hamburg, Germany). cDNA was synthesized by reverse transcription with an Advantage RT-for-PCR Kit (Takara Bio Inc., Otsu, Shiga, Japan). Realtime PCR was performed using SYBR Premix ExTaq II (Takara Bio Inc.) with a DICE thermal cycler (Takara Bio Inc.). *Gapdh* expression served as internal control.

Primer sequences were:

- *Gapdh forward: 5′-*ACCCAGAAGACTGTGGATGG*-3′.*
  *Gapdh reverse: 5′-*TTCAGCTCTGGGATGACCTT*-3′.*
  *Atrogin-1 forward: 5′-*GAGACCATTCTACACTGGCAGCA*-3′.*
  *Atrogin-1 reverse: 5′-*GTCACTCAGCCTCTGCATGATGT*-3′.*
  *MuRF1 forward: 5′-*ACCTGCTGGTGGAAAACATCATT*-3′.*
  *MuRF1 reverse: 5′-*AGGAGCAAGTAGGCACCTCACAC*-3′.*
  *Tnfα forward: 5′-*CTTCTGTCTACTGAACTTCGGG*-3′.*
  *Tnfα reverse: 5′-*CAGGCTTGTCACTCGAATTTTG*-3′.*
  *Il-1β forward: 5′-*AAGTTGACGGACCCCAAAAGAT*-3′.*
  *Il-1β reverse: 5′-*AGCTCTTGTTGATGTGCTGCTG*-3′.*
  *Il-6 forward: 5′-*GTCCTTAGCCACTCCTTCTG*-3′.*
  *Il-6 reverse: 5′-*CAAAGCCAGAGTCCTTCAGAG*-3′.*

### Western blotting

Lysates were obtained from frozen minced gastrocnemius muscle using RIPA buffer (1% Tween 20, 0.1% SDS, 150 nM NaCl, 10 mM Tris–HCl (pH 7.4), 1 mM phenylmethylsulfonyl fluoride (#P7626, Sigma-Aldrich Co. LLC, St. Louis, MO), 50 μg/ml aprotinin (#A1153, Sigma-Aldrich Co. LLC), 100 μg/ml leupeptin (#L2884, Sigma-Aldrich Co. LLC), 1 mM Na_3_VO_4_ (198-09752, FUJIFILM Wako Pure Chemical Corporation, Osaka, Japan), 25 μM pepstatin A (#P5318, Sigma-Aldrich Co. LLC)). 30 μg protein was loaded onto and run on 12.5% SDS–PAGE gels (e-PAGEL, ATTO Corporation, Tokyo, Japan) and then transferred to PolyVinylidene DiFluoride (PVDF) membranes (Immobilon, Merck KGaA, Darmstadt, Germany). Membranes were blocked with buffer containing 10 mM Tris·HCl (pH 7.4), 150 mM NaCl, 0.1% Tween 20, and 5% skim milk or bovine serum albumin and then incubated with each primary antibody overnight at 4 °C. Membranes were then incubated with HRP-conjugated Goat anti-Rabbit IgG (1:5,000; G21234, Thermo Fisher Scientific, Waltham, MA) as secondary antibody, and immune complexes were visualized using the ECL Western Blotting Analysis System (RPN2235, GE Healthcare, Chicago, IL).

Primary antibodies used to detect proteins were anti-phospho-Smad2 (1:1,000; #3,101, Cell Signaling Technology, Inc., Beverly, MA), anti-phospho-Smad3 (1:1,000; #9,520, Cell Signaling), anti-Smad2/3 (1:1,000; #3,102, Cell Signaling) and anti-Gapdh (1:20,000; GTX100118, GeneTex, Irvine, CA). ImageJ v. 1.51 software (National Institutes of Health, Bethesda, MD) was used to quantify each band.

### Statistical analysis

Data are shown as means ± SD. Statistical significance was assessed using Student’s t-test. A probability of less than 5% was considered statistically significant (*P < 0.05; **P < 0.01; ***P < 0.001; ns, not significant).

## Supplementary information

Supplementary information.

## References

[1] Cruz-Jentoft AJ (2010). Sarcopenia: European consensus on definition and diagnosis: Report of the European Working Group on Sarcopenia in Older People. Age Ageing.

[2] Conzade R (2019). Vitamin D in relation to incident sarcopenia and changes in muscle parameters among older adults: the KORA-age study. Calcif. Tissue Int..

[3] Scott D, Blizzard L, Fell J, Ding C, Winzenberg T, Jones G (2010). A prospective study of the associations between 25-hydroxy-vitamin D, sarcopenia progression and physical activity in older adults. Clin. Endocrinol..

[4] Cummings SR (1993). Bone density at various sites for prediction of hip fractures. The Study of Osteoporotic Fractures Research Group. Lancet.

[5] Odvina CV, Wergedal JE, Libanati CR, Schulz EE, Baylink DJ (1988). Relationship between trabecular vertebral body density and fractures: a quantitative definition of spinal osteoporosis. Metabolism.

[6] Bischoff-Ferrari HA (2004). Effect of vitamin D on falls: a meta-analysis. JAMA.

[7] Murad MH (2011). Clinical review: the effect of vitamin D on falls: a systematic review and meta-analysis. J. Clin. Endocrinol. Metab..

[8] Wu H, Pang Q (2017). The effect of vitamin D and calcium supplementation on falls in older adults: a systematic review and meta-analysis. Der. Orthopad..

[9] Tricco AC (2017). Comparisons of interventions for preventing falls in older adults: a systematic review and meta-analysis. JAMA.

[10] Greenspan SL, Myers ER, Maitland LA, Resnick NM, Hayes WC (1994). Fall severity and bone mineral density as risk factors for hip fracture in ambulatory elderly. JAMA.

[11] Arnstein AR, Frame B, Frost HM (1967). Recent progress in osteomalacia and rickets. Ann. Intern. Med..

[12] Hughes MR (1988). Point mutations in the human vitamin D receptor gene associated with hypocalcemic rickets. Science.

[13] Yoshizawa T (1997). Mice lacking the vitamin D receptor exhibit impaired bone formation, uterine hypoplasia and growth retardation after weaning. Nat. Genet..

[14] Pike JW, Meyer MB, Lee SM, Onal M, Benkusky NA (2017). The vitamin D receptor: contemporary genomic approaches reveal new basic and translational insights. J. Clin. Investig..

[15] Hou YC (2018). Role of nutritional vitamin D in osteoporosis treatment. Clin. Chim. Acta.

[16] Kitazawa R, Kitazawa S, Maeda S (1999). Promoter structure of mouse RANKL/TRANCE/OPGL/ODF gene. Biochem. Biophys. Acta.

[17] Hofbauer LC, Dunstan CR, Spelsberg TC, Riggs BL, Khosla S (1998). Osteoprotegerin production by human osteoblast lineage cells is stimulated by vitamin D, bone morphogenetic protein-2, and cytokines. Biochem. Biophys. Res. Commun..

[18] Sato Y (2014). The vitamin D analogue ED71 but Not 1,25(OH)2D3 targets HIF1alpha protein in osteoclasts. PLoS ONE.

[19] Takasu H (2006). c-Fos protein as a target of anti-osteoclastogenic action of vitamin D, and synthesis of new analogs. J. Clin. Investig..

[20] Lieben L (2012). Normocalcemia is maintained in mice under conditions of calcium malabsorption by vitamin D-induced inhibition of bone mineralization. J. Clin. Investig..

[21] Hao W (2015). Hyperglycemia promotes schwann cell de-differentiation and de-myelination via sorbitol accumulation and igf1 protein down-regulation. J. Biol. Chem..

[22] Sommer L, Suter U (1998). The glycoprotein P0 in peripheral gliogenesis. Cell Tissue Res..

[23] D'Urso D (1990). Protein zero of peripheral nerve myelin: biosynthesis, membrane insertion, and evidence for homotypic interaction. Neuron.

[24] Pojednic RM (2015). Effects of 1,25-dihydroxyvitamin D3 and vitamin D3 on the expression of the vitamin d receptor in human skeletal muscle cells. Calcif. Tissue Int..

[25] Cruz-Jentoft AJ, Sayer AA (2019). Sarcopenia. Lancet.

[26] Michael K (2000). Relationship of skeletal muscle atrophy to functional status: a systematic research review. Biol. Res. Nurs..

[27] Oikawa SY, Holloway TM, Phillips SM (2019). The impact of step reduction on muscle health in aging: protein and exercise as countermeasures. Front. Nutr..

[28] Tando T (2016). Smad2/3 proteins are required for immobilization-induced skeletal muscle atrophy. J. Biol. Chem..

[29] Johnson JA, Grande JP, Windebank AJ, Kumar R (1996). 1,25-Dihydroxyvitamin D(3) receptors in developing dorsal root ganglia of fetal rats. Brain Res. Dev. Brain Res..

[30] Cornet A, Baudet C, Neveu I, Baron-Van Evercooren A, Brachet P, Naveilhan P (1998). 1,25-Dihydroxyvitamin D3 regulates the expression of VDR and NGF gene in Schwann cells in vitro. J. Neurosci. Res..

[31] Tague SE, Smith PG (2011). Vitamin D receptor and enzyme expression in dorsal root ganglia of adult female rats: modulation by ovarian hormones. J. Chem. Neuroanat..

[32] Le Douarin NM, Smith J (1988). Development of the peripheral nervous system from the neural crest. Annu. Rev. Cell Biol..

[33] Mayor R, Theveneau E (2013). The neural crest. Development.

[34] Schakman O, Kalista S, Barbe C, Loumaye A, Thissen JP (2013). Glucocorticoid-induced skeletal muscle atrophy. Int. J. Biochem. Cell Biol..

[35] Nakamura H (1982). Mesenchymal derivatives from the neural crest. Arch. Histol. Japonicum.

[36] Holick MF (2007). Vitamin D deficiency. N. Engl. J. Med..

[37] Soliman AT, Al Khalaf F, Alhemaidi N, Al Ali M, Al Zyoud M, Yakoot K (2008). Linear growth in relation to the circulating concentrations of insulin-like growth factor I, parathyroid hormone, and 25-hydroxy vitamin D in children with nutritional rickets before and after treatment: endocrine adaptation to vitamin D deficiency. Metabolism.

[38] Sakai S (2015). Vitamin D receptor signaling enhances locomotive ability in mice. J. Bone Mineral Res..

[39] Hijikawa T (2008). Insulin-like growth factor 1 prevents liver injury through the inhibition of TNF-alpha and iNOS induction in D-galactosamine and LPS-treated rats. Shock.

[40] Sukhanov S (2007). IGF-1 reduces inflammatory responses, suppresses oxidative stress, and decreases atherosclerosis progression in ApoE-deficient mice. Arterioscler. Thromb. Vasc. Biol..

[41] Matsuoka LY, Wortsman J, Hollis BW (1990). Use of topical sunscreen for the evaluation of regional synthesis of vitamin D3. J. Am. Acad. Dermatol..

[42] Libon F (2017). Sunscreens block cutaneous vitamin D production with only a minimal effect on circulating 25-hydroxyvitamin D. Arch. Osteop..

[43] Holick MF (2006). High prevalence of vitamin D inadequacy and implications for health. Mayo Clin. Proc..

[44] Mithal A (2009). Global vitamin D status and determinants of hypovitaminosis D. Osteop. Int..

[45] Bartoszewska M, Kamboj M, Patel DR (2010). Vitamin D, muscle function, and exercise performance. Pediatr. Clin. N. Am..

[46] Nakamura K, Nashimoto M, Matsuyama S, Yamamoto M (2001). Low serum concentrations of 25-hydroxyvitamin D in young adult Japanese women: a cross sectional study. Nutrition.

[47] Miyamoto T (2016). Vitamin D deficiency with high intact PTH levels is more common in younger than in older women: a study of women aged 39–64 years. Keio J. Med..

[48] Li YC (1998). Normalization of mineral ion homeostasis by dietary means prevents hyperparathyroidism, rickets, and osteomalacia, but not alopecia in vitamin D receptor-ablated mice. Endocrinology.

[49] Bischoff-Ferrari HA (2006). Is fall prevention by vitamin D mediated by a change in postural or dynamic balance?. Osteop. Int..

[50] Muir SW, Montero-Odasso M (2011). Effect of vitamin D supplementation on muscle strength, gait and balance in older adults: a systematic review and meta-analysis. J. Am. Geriatr. Soc..

[51] Saito K, Miyakoshi N, Matsunaga T, Hongo M, Kasukawa Y, Shimada Y (2016). Eldecalcitol improves muscle strength and dynamic balance in postmenopausal women with osteoporosis: an open-label randomized controlled study. J. Bone Miner. Metab..

[52] Matsumoto T (2012). Osteoporosis treatment by a new active vitamin D3 compound, eldecalcitol, Japan. Curr. Osteop. Rep..

[53] Noguchi Y, Kawate H, Nomura M, Takayanagi R (2013). Eldecalcitol for the treatment of osteoporosis. Clin. Interv. Aging.

[54] Srikuea R, Zhang X, Park-Sarge OK, Esser KA (2012). VDR and CYP27B1 are expressed in C2C12 cells and regenerating skeletal muscle: potential role in suppression of myoblast proliferation. Am. J. Physiol. Cell Physiol..

[55] Girgis CM (2014). The vitamin D receptor (VDR) is expressed in skeletal muscle of male mice and modulates 25-hydroxyvitamin D (25OHD) uptake in myofibers. Endocrinology.

[56] Tanaka K, Kanazawa I, Yamaguchi T, Yano S, Kaji H, Sugimoto T (2014). Active vitamin D possesses beneficial effects on the interaction between muscle and bone. Biochem. Biophys. Res. Commun..

[57] Saito H, Kishimoto KN, Mori Y, Okuno H, Tanaka M, Itoi E (2017). A vitamin D analogue, eldecalcitol, enhances expression of fast myosin heavy chain subtypes in differentiated C2C12 myoblasts. J Orthop. Sci..

[58] Nakao R (2009). Ubiquitin ligase Cbl-b is a negative regulator for insulin-like growth factor 1 signaling during muscle atrophy caused by unloading. Mol. Cell. Biol..

[59] Kawai N (2015). Prevention of skeletal muscle atrophy in vitro using anti-ubiquitination oligopeptide carried by atelocollagen. Biochem. Biophys. Acta..

[60] Hofmann M (2015). Serum concentrations of insulin-like growth factor-1, members of the TGF-beta superfamily and follistatin do not reflect different stages of dynapenia and sarcopenia in elderly women. Exp. Gerontol..

[61] Nakamura S (2019). Insulin-like growth factor-I is required to maintain muscle volume in adult mice. J. Bone Miner. Metab..

[62] van Deursen RW, Simoneau GG (1999). Foot and ankle sensory neuropathy, proprioception, and postural stability. J. Orthop. Sports Phys Therapy.

[63] Ponchon G, Kennan AL, DeLuca HF (1969). "Activation" of vitamin D by the liver. J. Clin. Investig..

[64] Bouillon R, Carmeliet G (2018). Vitamin D insufficiency: Definition, diagnosis and management. Best Pract. Res. Clin. Endocrinol. Metab..

[65] DeLuca HF (2004). Overview of general physiologic features and functions of vitamin D. Am. J. Clin. Nutr..

[66] Watanabe R (2017). Enpp1 is an anti-aging factor that regulates Klotho under phosphate overload conditions. Sci. Rep..

[67] Yamamoto Y (2013). Vitamin D receptor in osteoblasts is a negative regulator of bone mass control. Endocrinology.

