# Supplementary material for: Vitamin D protects against immobilization-induced muscle atrophy via neural crest-derived cells in mice
Source: Sci Rep. 2020 Jul 22;10:12242. doi: 10.1038/s41598-020-69021-y (PMC7376070; doi:10.1038/s41598-020-69021-y)

Vitamin D protects against immobilization-induced muscle atrophy via neural crest-derived cells in mice

Satoshi Nakamura[1], Yuiko Sato[1,2,3], Tami Kobayashi[1,3], Yosuke Kaneko[1], Eri Ito[4], Tomoya Soma[5], Hiroyuki Okada[6], Kana Miyamoto[1], Akihito Oya[1], Morio Matsumoto[1], Masaya Nakamura[1], Arihiko Kanaji[1] and Takeshi Miyamoto[1, 2,3, 7*]

[1]Department of Orthopedic Surgery, [2]Department of Advanced Therapy for Musculoskeletal Disorders II, [3]Department of Musculoskeletal Reconstruction and Regeneration Surgery, [4]Institute for Integrated Sports Medicine, [5]Division of Oral and Maxillofacial Surgery, Department of Dentistry and Oral Surgery, Keio University School of Medicine, 35 Shinano-machi, Shinjuku-ku, Tokyo 160-8582, Japan, [6]Department of Orthopedic Surgery, The University of Tokyo, 7-3-1 Hongo, Bunkyo-ku, Tokyo 113-0033, Japan, [7]Department of Orthopedic Surgery, Kumamoto University, 1-1-1 Honjo, Chuo-ku, Kumamoto 860-8556, Japan

Supplementary Materials and Methods

Immunohistochemistry

Sciatic nerve or gastrocnemius muscle tissues were embedded in paraffin and cut into 4 µm sections. Samples were blocked in 5 % bovine serum albumin/PBS containing 0.1 % Tween 20 for 1 hour at room temperature and then incubated with respective primary antibodies overnight at 4 °C. Antibodies used were anti-Vitamin D receptor (1:100; ab8756, Abcam, Cambridge, UK) or anti-Myelin Protein Zero (1:100; ab31851, abcam) Then, after three PBS washes, goat anti-rabbit Alexa Fluor 488 (1:200; Invitrogen, Waltham, MA) and goat anti-rat Alexa Fluor 546 (1:200; Invitrogen) were added as secondary antibodies for 1 hour at room temperature. DAPI (1:5000; Wako Pure Chemicals Industries, Osaka, Japan) served as a nuclear stain.

Blood analysis

Sera were collected just before sacrifice. Serum cortisol levels were measured by ECLIA. Serum PTH levels were analyzed with Mouse PTH 1-84 ELISA Kit (Quidel Corporation,

San Diego, CA) based on standard procedures.

Western blotting

Lysates were obtained from frozen sciatic nerve minced in RIPA buffer (1% Tween 20, 0.1% SDS, 150 nM NaCl, 10 mM Tris-HCl (pH 7.4), 1 mM phenylmethylsulfonyl fluoride, 50 μg/ml aprotinin, 100 μg/ml leupeptin, 1 mM Na_3VO_4, 25 μM pepstatin A). 12 μg protein was loaded and run on 12.5% SDS–PAGE gels and transferred to PVDF membranes. Membranes were blocked with buffer containing 10 mM Tris·HCl (pH 7.4), 150 mM NaCl, 0.1% Tween 20, and 5% skim milk or bovine serum albumin and then incubated with each primary antibody overnight at 4 °C. Primary antibodies used were anti-IGF-1 (1:1000; bs-0014R, Bioss Antibodies, Woburn, MA) or anti-Gapdh (1:20000). After washing, membranes were then incubated with HRP-conjugated Goat anti-Rabbit IgG (1:5000; G21234, Thermo Fisher Scientific, Waltham, MA) secondary antibody, and immune complexes were visualized using the ECL Western Blotting Analysis System (RPN2235, GE Healthcare, Chicago, IL).

Supplementary Figure Legends

Figure S1. Schemas showing experimental protocols using in Fig. 2.

Schemas showing protocols used in Fig. 2b-c (a), Fig. 2d-e (b), and Fig. 2f-I (c).

Figure S2. sVDR cKO and mVDR cKO knockout efficiently.

Neural crest-specific sVDR cKO (P0Cre;*VDR*$^{flox/flox}$), skeletal muscle-specific mVDR KO (CkmmCre;*Vdr*$^{flox/flox}$) and control (*Vdr*$^{flox/flox}$) female mice were fed the S diet, their hind limbs were stapled at 9 weeks of age, and animals were sacrificed 1 week later. (a) Double immunohistochemistry for P0 (green) and VDR (red) in sciatic nerve on the non-stapled side of sVDR cKO and *Vdr*$^{flox/flox}$ animals. P0 (green) and VDR (red) staining is lost in sciatic nerve of sVDR cKO relative to *Vdr*$^{flox/flox}$ samples. (b) Gastrocnemius muscle on the non-stapled side of mVDR cKO and *Vdr*$^{flox/flox}$ samples stained with laminin (green) and VDR (red). VDR staining is decreased in gastrocnemius muscle of mVDR cKO relative to *Vdr*$^{flox/flox}$ samples. DAPI (blue) served as a nuclear stain in both cases. Scale bar, 100 μm.

Figure S3. Serum Cortisol or PTH levels are comparable in sVDR cKO and control mice.

(a and b) Neural crest-specific sVDR cKO and control (*Vdr*$^{flox/flox}$) female mice were fed the S diet, their hind limbs were stapled at 9 weeks of age, and animals were sacrificed 1 week later (n = 5 per group). Sera were collected immediately before sacrifice, and levels of cortisol (a) or PTH (b) analyzed by ELISA. Data represent mean serum levels of either in sVDR cKO relative to *VDR*$^{flox/flox}$ mice ± SD. Statistical analysis was performed using Student's t-test (*P < 0.05; **P < 0.01; ***P<0.001; ns, not significant).

Figure S4. Administration of an active vitamin D analogue to sVDR cKO mice cannot rescue immobilization-induced muscle atrophy phenotypes.

Neural crest-specific sVDR cKO female mice were fed the S diet, and treated with 3.5 ng ED71 or vehicle (n = 5 per group) twice per week starting at 8 weeks of age. Their hind limbs were stapled a week later, and mice were sacrificed 1 week after that. Wet weights of gastrocnemius (a) and quadriceps (b) muscle adjusted to body weight in Veh and ED

groups were determined. Shown is mean muscle weight relative to the control side of the Veh group ± SD. Statistical analysis was performed using Student's t-test (*P < 0.05; **P < 0.01; ***P<0.001; ns, not significant).

Figure S5. IGF-1 expression decreases in sciatic nerve from sVDR cKO mice.

Neural crest-specific sVDR cKO and control (*Vdr*$^{flox/flox}$) female mice were fed the S diet, their hind limbs were stapled at 9 weeks of age, and animals were sacrificed 1 week later. Shown is IGF-1 expression in sciatic nerve from sVDR cKO and *Vdr*$^{flox/flox}$ mice, as detected by western blot. Gapdh serves as an internal control.

Figure S6. Full-length western blots.

Full-length western blots of Fig. 1i (a-c), Fig. 2o (d-f) and Fig. S5 (g and h). First antibodies were anti-pSmad2 and anti-pSmad3 (a and d), anti-Smad2/3 (b and e), anti-IGF-1 (g) and anti-Gapdh (c, f and h). Arrows showed bands of indicated proteins.

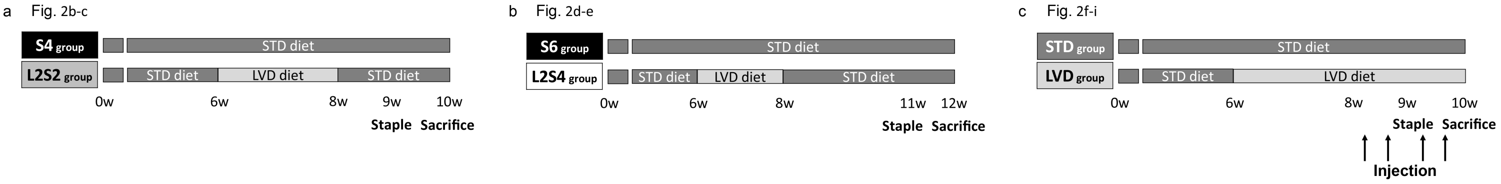
a
Fig. 2b-c
S4 group
STD diet
L2S2 group
STD diet
LVD diet
STD diet
0w
6w
8w
9w
10w
Staple
Sacrifice
b
Fig. 2d-e
S6 group
STD diet
L2S4 group
STD diet
LVD diet
STD diet
0w
6w
8w
11w
12w
Staple
Sacrifice
c
Fig. 2f-i
STD group
STD diet
LVD group
STD diet
LVD diet
0w
6w
8w
9w
10w
Staple
Sacrifice
Injection

a

b

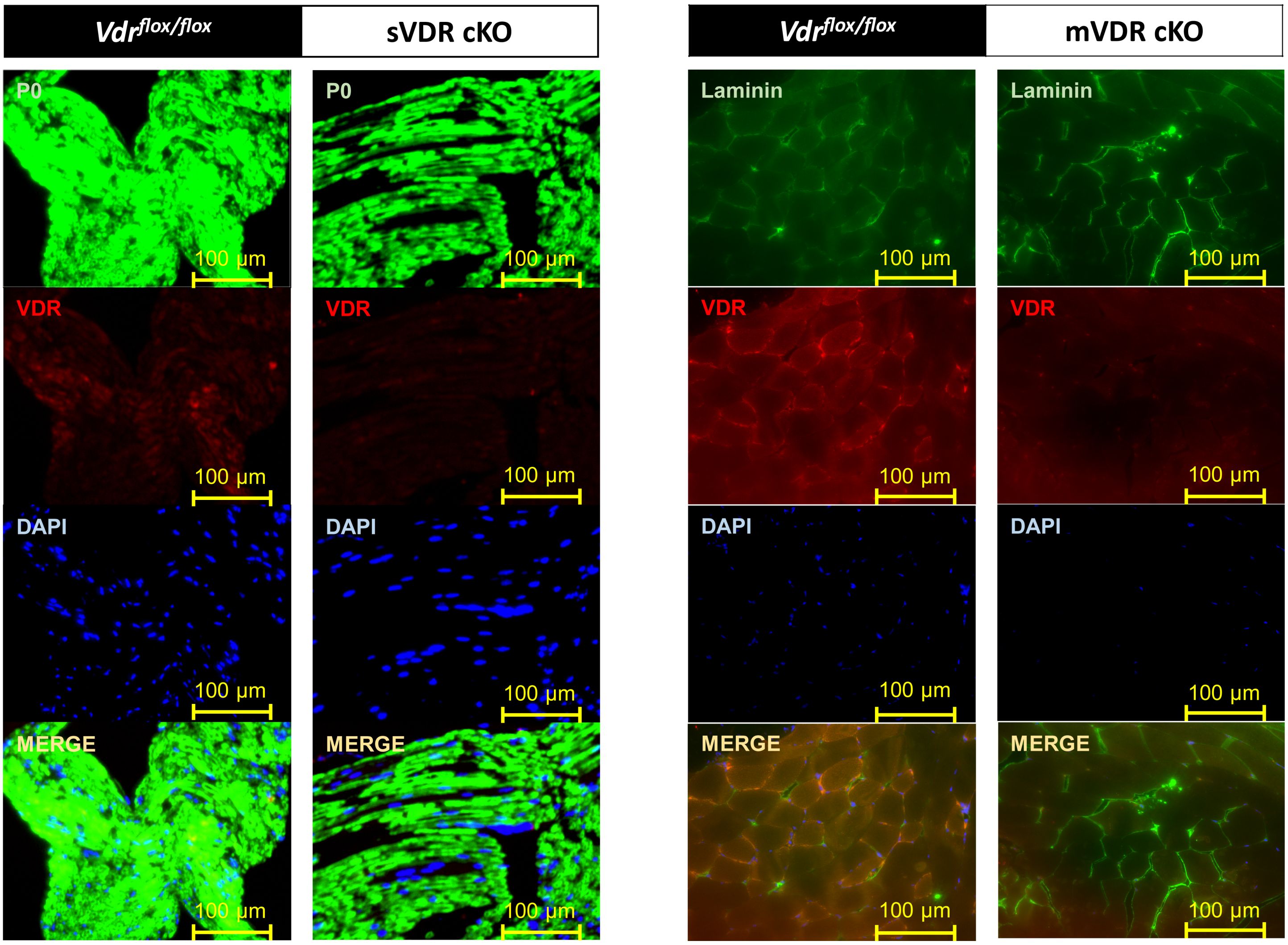
Vdrflox/flox
sVDR cKO
P0
VDR
DAPI
MERGE
100 μm
Vdrflox/flox
mVDR cKO
Laminin
VDR
DAPI
MERGE
100 μm

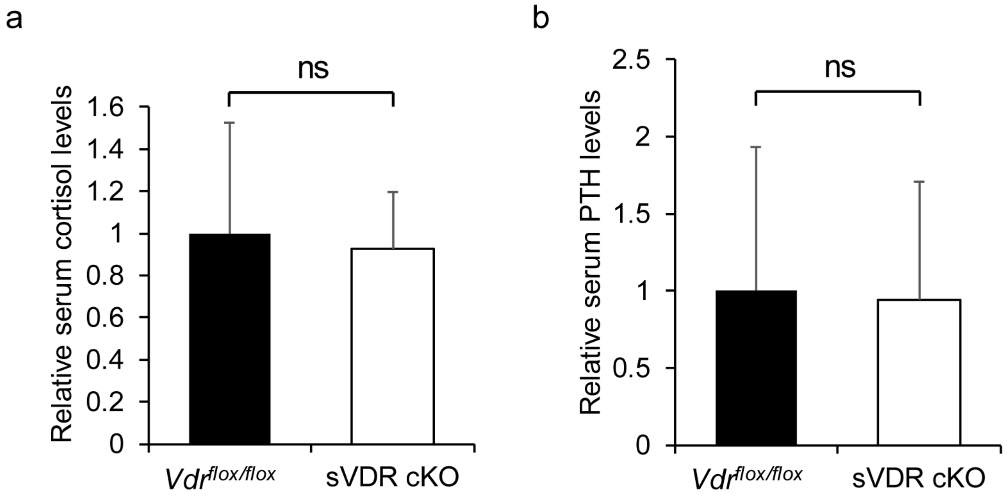
a
b
ns
ns
Relative serum cortisol levels
Relative serum PTH levels
1.6
1.4
1.2
1
0.8
0.6
0.4
0.2
0
2.5
2
1.5
1
0.5
0
Vdr^flox/flox
sVDR cKO
Vdr^flox/flox
sVDR cKO

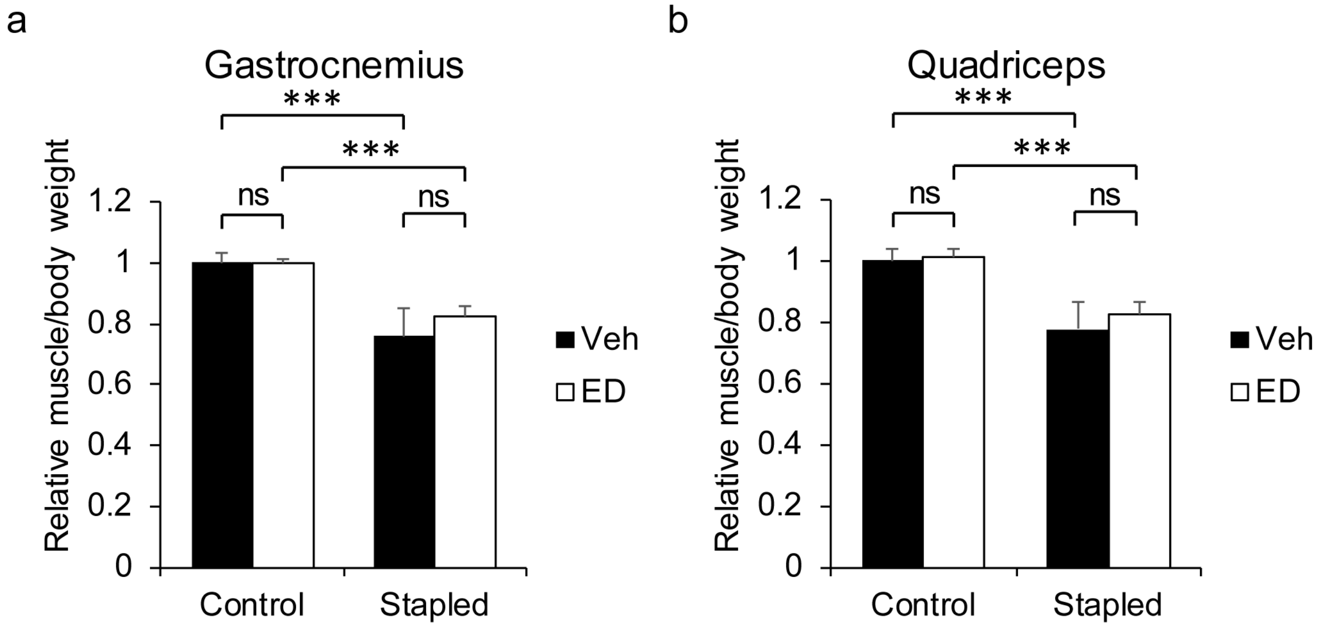
a
Gastrocnemius
***
***
ns
ns
1.2
1
0.8
0.6
0.4
0.2
0
Relative muscle/body weight
Control
Stapled
Veh
ED
b
Quadriceps
***
***
ns
ns
1.2
1
0.8
0.6
0.4
0.2
0
Relative muscle/body weight
Control
Stapled
Veh
ED

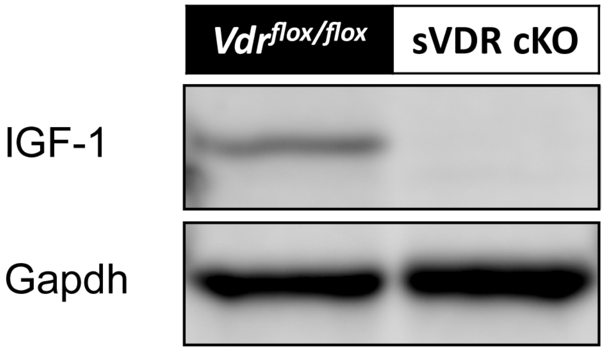
Vdr flox/flox
sVDR cKO
IGF-1
Gapdh

Figure 1i

a pSmad2, pSmad3

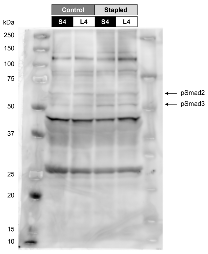

b Smad2, Smad3

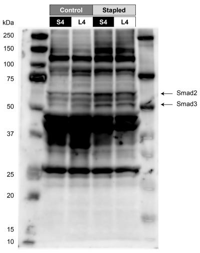

c Gapdh

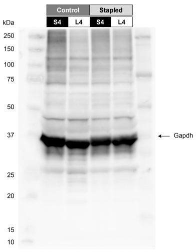

Figure 2o

d pSmad2, pSmad3

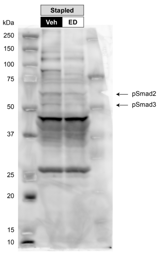

e Smad2, Smad3

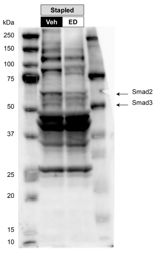

f Gapdh

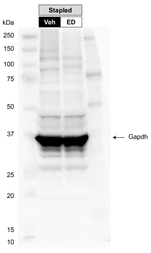

Figure S5

g IGF-1

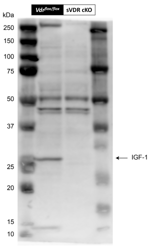

h Gapdh

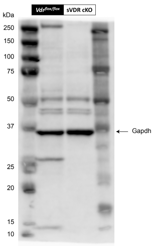

Supplement: Supplementary file 1 — Supplementary information. [file 41598_2020_69021_MOESM1_ESM.pdf]
